# The Effect of C-Reactive Protein Isoforms on Nitric Oxide Production by U937 Monocytes/Macrophages

**DOI:** 10.3389/fimmu.2018.01500

**Published:** 2018-07-02

**Authors:** Nicola R. Sproston, Mohamed El Mohtadi, Mark Slevin, William Gilmore, Jason J. Ashworth

**Affiliations:** School of Healthcare Science, Manchester Metropolitan University, Manchester, United Kingdom

**Keywords:** C-reactive protein, nitric oxide, inflammation, native C-reactive protein, monomeric C-reactive protein, estrogen

## Abstract

Inflammation is regulated by many endogenous factors including estrogen, a steroid hormone that declines with increasing age, leading to excessive inflammation in the elderly. C-reactive protein (CRP) is an acute phase inflammatory protein that exists in two forms, native CRP (nCRP) and monomeric CRP (mCRP), which mediate distinct biological activities. It is unclear how each CRP isoform mediates nitric oxide (NO), a signaling molecule generated by NO synthase (NOS). This study investigated whether CRP isoforms have distinct effects on NO production by unstimulated and lipopolysaccharide (LPS)-activated monocytes/macrophages and whether estrogen mediates CRP-induced NO production in an *in vitro* model of aging. NO and inducible NOS (iNOS) were measured (*n* = 12) by the Griess assay and an enzyme-linked immunosorbent assay, respectively following incubation (24 h) of human-derived U937 monocytes/macrophages with CRP isoforms [(nCRP) = 500 and 1,000 µg/ml; (mCRP) = 100 and 250 µg/ml] in the absence or presence of 17 beta-estradiol (1 × 10^−7^, 1 × 10^−8^, and 1 × 10^−9^ M). The response to each CRP isoform and estrogen was dependent on the differentiation and activation status of cells. Monocytes with or without prior LPS-activation significantly increased (*P* < 0.01) NO/iNOS production when treated with mCRP. The mCRP isoform had no effect (*P* > 0.05) on NO/iNOS production by unactivated or LPS-activated macrophages, whereas nCRP significantly (*P* < 0.05) reduced NO/iNOS production by macrophages, with or without prior LPS-activation. The nCRP isoform had opposing actions on monocytes, significantly (*P* < 0.01) increasing and reducing NO/iNOS by unactivated and LPS-activated monocytes, respectively. Estrogen significantly (*P* < 0.01) reversed nCRP-mediated NO inhibition by unactivated macrophages but decreased CRP-induced NO by unactivated monocytes treated with nCRP or mCRP and LPS-activated monocytes treated with mCRP. NO was differentially mediated by CRP isoforms in a cell-type/state-specific manner, with production corresponding to concomitant changes in iNOS levels. Collectively, the findings indicate nCRP and estrogen predominantly reduce NO production, whereas mCRP increases NO production. This supports growing evidence that mCRP exacerbates inflammation while nCRP and estrogen dampen the overall inflammatory response. Therapeutic strategies that restore estrogen levels to those found in youth and promote the stability of nCRP or/and prevent the formation of mCRP may reduce NO production in age-related inflammatory conditions.

## Introduction

There is increasing evidence that C-reactive protein (CRP) may play a role in inflammatory processes. CRP is a homopentameric acute-phase inflammatory protein that exhibits elevated expression during inflammatory conditions, such as rheumatoid arthritis, some cardiovascular diseases, and infection (1). The pentameric protein, termed native CRP (nCRP) is characterized by a discoid configuration of five identical non-covalently bound subunits, each 206 amino acids long with a molecular mass of about 23 kDa (1).

The nCRP isoform is synthesized primarily in liver hepatocytes but has also been reported to be synthesized in other cell types, such as smooth muscle cells (2), macrophages (3), endothelial cells (4), lymphocytes (5), and adipocytes (6). CRP is first synthesized as monomers and then assembled into the pentamer in the endoplasmic reticulum of the cell. The stimulation of CRP synthesis mainly occurs in response to pro-inflammatory cytokines, most notably interleukin-6 (IL-6), IL-1, and tumor necrosis factor-alpha (TNF-α) (7,8).

Pentameric CRP can irreversibly dissociate, with the resultant-free subunits termed monomeric (or modified) CRP (mCRP). The mCRP isoform is distinguished from nCRP by its distinct antigenic, biological, and electrophoretic activities (9) and the fact it expresses different neoepitopes (10). Evidence suggests nCRP which often exhibits a more anti-inflammatory function in tissues relative to the mCRP isoform. It is suggested that this occurs by nCRP binding at sites of tissue injury to limit the generation of the membrane attack complex (MAC) and C5a, thus inhibiting complement activation (11). On the other hand, mCRP generally has marked pro-inflammatory properties *in vitro* and *in vivo* by promoting the recruitment of circulating leukocytes to areas of inflammation through interaction with receptors of the Fcγ family (12) and lipid raft microdomains in cell membranes (13).

There has been some evidence that CRP interacts with nitric oxide (NO), a short-lived, pleiotropic-free radical regulator of various biological functions, including vasodilation, neurotransmission, inflammation, and macrophage-mediated immunity (14). NO has properties that allow it to be easily soluble and diffuse across biological membranes to conduct its intracellular processes (15). This intercellular signaling molecule is important for the immune system, as well as generating free oxygen radicals called peroxynitrites (ONOO^−^) which act in a cytotoxic manner causing tissue damage and apoptosis (16). NO also plays a functional role in processes, such as leukocyte adhesion and transmigration, proliferation, apoptosis, and cytokine expression (17–20). NO is generated by NO synthase (NOS) enzymes (21); neuronal NOS, constitutive eNOS, and inducible NOS (iNOS). NO production in inflammatory conditions and following infection is predominantly through iNOS (22).

The versatility of NO at inducing variable responses suggests it has both pro- and anti-inflammatory effects. However, the inhibition of NO pathways is often beneficial in the treatment of inflammatory disease (23). Several factors modify the effect of NO including its concentration, the rate of reactive nitrogen species formation and the physiological environment. During the inflammatory response, pro-inflammatory cytokines stimulate NO production in monocytes, macrophages, and neutrophils and *in vivo* levels can increase by up to 1,000-fold in severe cases of bacterial sepsis (22). NO acts as a positive feedback molecule when released by tissue macrophages during phagocytosis, leading to the recruitment of further phagocytes. However, excessive NO can lead to tissue destruction as seen in autoimmune diseases (24). The level of NO produced is regulated at the transcriptional level, depending on the cell type and the nature of the stimulation involved. The mitogen-activated protein kinase and PI3 kinase pathways mediate NO production (25) but there is conflicting evidence if the p38 pathway is involved, with studies showing upregulation, downregulation, and no effect on NO production (26, 27). NO can also regulate its own production *via* positive and negative feedback loops. The positive feedback utilizes the increase in cAMP levels to activate the production of iNOS and subsequent increase in NO production. The negative feedback loop uses the inhibition of NFκβ to lower NO production (25).

Several studies that have highlighted a relationship between NO production and CRP. Hattori et al. (28) showed CRP mediates both NO and iNOS gene expression. Schwedler et al. (21) found that nCRP treatment *in vivo* caused impairment in endothelial function in the aortic rings that was associated with increased iNOS activity. Several cardiovascular studies have revealed that nCRP inhibits NO production by downregulating endothelial NOS (eNOS) in endothelial cells, thereby inhibiting angiogenesis (29). Inhibition of endothelial-derived NO promotes the pathogenesis of atherosclerotic vascular disease through vasoconstriction, leukocyte adherence, and inflammatory cell activation (29–32), highlighting the pro-inflammatory nature of nCRP in the circulatory system. Little work has been published on the effect of CRP on NO production by cells and tissues outside the cardiovascular system. Borderie et al. (33) showed that CRP concentration correlates with the number of iNOS-positive synovial fluid leukocytes in patients with rheumatoid arthritis but specific CRP isoforms were not distinguished. The mCRP isoform has subsequently been shown to enhance NO production in isolated human neutrophils *via* upregulated NOS activity (34). Consequently, this study assessed whether CRP isoforms have distinct effects on iNOS and NO production by human-derived U937 monocytes/macrophages.

Nitric oxide is also regulated by many endogenous factors including the steroid hormone estrogen (35). Estrogen is known to have beneficial effects during wound repair following tissue injury, accelerating healing, and reducing inflammation in both male and female humans (36, 37). Moreover, the profound decline in estrogen levels in the elderly is implicated in age-related impaired healing, leading to delayed repair with excessive inflammation (38). Studies have shown that estrogen has the ability to stimulate NO production and vasodilation in the cardiovascular system by inducing eNOS (39). In contrast, Hassouna et al. (40) showed that the loss of estrogen following ovariectomy in a rat model of inflammation significantly increased levels of both CRP and iNOS, while subsequent estrogen supplementation reversed this effect. This study investigated whether exogenous estrogen can mediate CRP-induced NO production by human-derived U937 monocytes/macrophages in an *in vitro* model of aging.

## Materials and Methods

### Purification of CRP Isoforms

The CRP isoforms were purified from a high purity commercial source of human nCRP (MBS536586; MyBioSource, San Diego, CA, USA). Purified mCRP was generated using the Potempa method (41). In this method, 1 ml commercial CRP protein was chelated in a 1:1 ratio with EDTA/urea buffer (10 mM EDTA, 8 M ultrapure urea) and incubated at 37°C for 2 h prior to dialysis (20 kDa MWCO) in buffer (25 mM Tris–HCl, 50 mM NaCl; pH 8.3) for 24 h.

Commercial nCRP (1 ml) was purified further by hydrophobic interaction chromatography using a Sepharose HiTrap column (GE Healthcare, Buckinghamshire) according to the manufacturer’s instructions. The eluted protein was immediately placed inside a 50 kDa MWCO float-a-lyzer (Medicell, London) and dialyzed overnight into a 2 mM calcium chloride buffer (2 mM CaCl_2_, 25 mM Tris–HCl, 150 mM NaCl; pH 7.4).

Both the nCRP and mCRP samples were tested for purity by immunoblotting using published methods (42). The immunoblotting procedure consisted of placing 5 µl of purified CRP samples onto two separate nitrocellulose membranes. A similar volume blot (5 µl) of 1 mg/ml bovine serum albumin (BSA) was used as a negative control protein and 5 µl of the original commercial CRP at 5 mg/ml was used as a positive control on each membrane. The samples were allowed to dry before placing the membranes in blocking buffer (1% BSA in TBS-Tween, pH 7.4) for 1 h at room temperature with 60 rpm rotation. Monoclonal mouse anti-human nCRP 1D6 [clone I-15-1D6 (isotype: IgG2a, κ)] and monoclonal mouse anti-human mCRP 8C10 [clone III-26-8C10 (IgG1, κ)] were primary antibodies kindly provided by L.A. Potempa (Roosevelt University, IL, USA; ImmTech Inc.) to detect specific CRP isoforms (43). Membranes were incubated overnight at 5°C with 50 rpm rotation in primary antibody diluted 1:10 with blocking buffer before washing five times in TBS-Tween buffer for 5 min. The membranes were then incubated in a 1:1,000 dilution of horseradish peroxidase (HRP)-conjugated polyclonal rabbit anti-mouse immunoglobulins (secondary antibody) (P0260; Dako, Cambridge) in 5% milk/TBS-tween solution for 1 h with 60 rpm rotation. The membranes were washed again five times in TBS-Tween buffer for 5 min. The membranes were then treated with the EZ-ECL chemiluminescence detection kit for HRP (K1-0172; Geneflow, Lichfield) according to the manufacturer’s instructions. The chemiluminescent image was then captured in a Syngene GBox with a 1 min exposure time using Genesnap software (Version 7.07, Syngene).

Purified CRP samples were quantified using bicinchronic acid (BCA) analysis (BCA1-1KT; Sigma-Aldrich, Poole) according to the manufacturer’s instructions to determine the yield. A standard curve was generated for the BCA analysis using a BSA standard. CRP samples were incubated with BCA working buffer for 30 min at 37°C prior to reading absorbance measurements at 595 nm on a Biotech Synergy HT plate reader. CRP concentrations were quantified by interpolation from the standard curve and then normalized at 2 mg/ml. Normalized CRP samples (2 mg/ml) were then sterile filtered and confirmed endotoxin-free using a commercial endotoxin detection E-TOXATE (*Limulus* amebocyte lysate) kit (ET0100; Sigma Aldrich, Poole) according to the manufacturer’s instructions. All CRP isoforms were stored at 4^o^C until use in cell assays.

### Cell Culture

**Figure 1:**
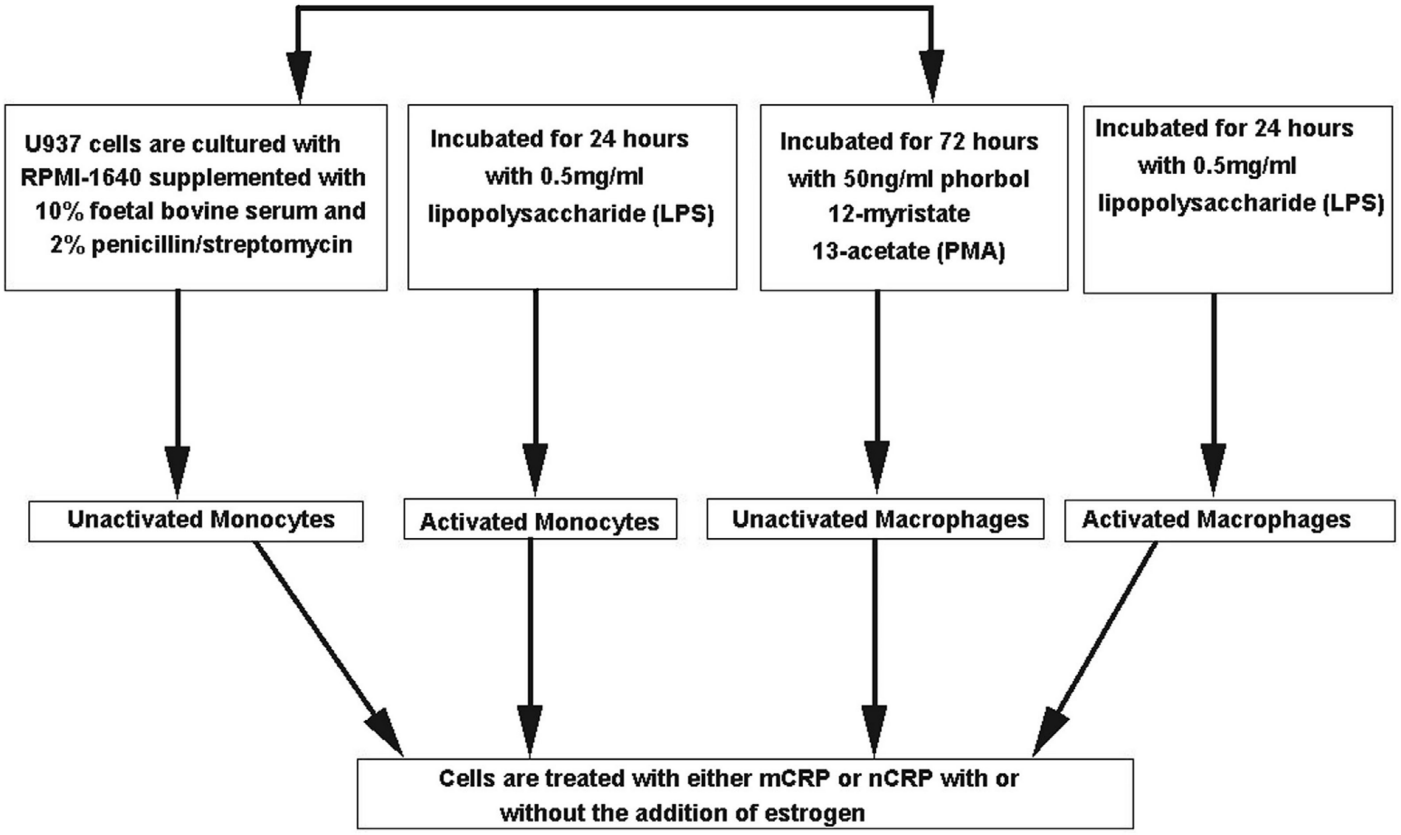
Flow chart of different treatment paths used to activate and differentiate the U937 cells. U937 cells were differentiated into a macrophage-like phenotype using 50 ng/ml phorbol 12-myristate 13-acetate (PMA) for 72 h and/or pre-activated for 24 h with 0.5 mg/ml lipopolysaccharide (LPS).

The monocyte-like cell line U937 was cultured in RPMI-1640 media supplemented with 10% FBS and 2% penicillin–streptomycin. In some experiments, U937 cells were differentiated into a macrophage-like phenotype using 50 ng/ml phorbol 12-myristate 13-acetate (PMA) for 72 h and/or pre-activated for 24 h with 0.5 mg/ml lipopolysaccharide (LPS). All cell culture experiments were performed in a class II safety cabinet according to local biosecurity and safety procedures. Figure 1 outlines the different treatment paths used to activate and differentiate the U937 cells. Cell viability was assessed using 0.4% trypan blue exclusion in which cellular uptake of the dye indicates cell death.

### Flow Cytometry

Differentiation of U937 monocytes into a macrophage-like phenotype was confirmed by flow cytometry through detection of the CD11c macrophage-specific surface antigen. Following differentiation with PMA, cells were treated with 4% paraformaldehyde in Dulbecco’s phosphate-buffered saline (DPBS) for 10 min at room temperature. Cells were washed twice with DPBS and stained for 30 min at room temperature with FITC-conjugated anti-human CD11c antibody (Clone Bu15; BioLegend, UK) diluted 1:40 with 10% fetal bovine serum (FBS) in DPBS. Cells were then washed twice in DPBS before suspension in 500 µL DPBS. CD11c expression was assessed on 10,000 events (live, individual cells) with a BD Accuri C6F1 cytometer using BD Accuri C6 software (Biosciences, USA) after gating in the forward scatter/side scatter and fluorescence parameter 1 (FL1-A) windows. Data were presented as average percentage CD11c^+^ cells (%) and median fluorescence intensity (MFI) relative to unstained monocytes from three independent experiments.

### NO and iNOS Assays

Cells at a concentration of 1 × 10^6^ cells/ml were incubated with physiological concentrations of mCRP (100 µg/ml or 250 µg/ml) or nCRP (500 µg/ml or 1,000 µg/ml) for 24 h. Negative controls were incubated with equivalent concentrations of BSA for 24 h. In inhibitor treatments, cells were co-incubated with CRP isoforms and the inhibitor nystatin at a concentration of 25 µg/ml. In the aging model, cells were co-incubated with CRP isoforms and estrogen at a concentration of 1 × 10^−7^, 1 × 10^−8^, and 1 × 10^−9^ M. Estrogen at 1 × 10^−8^ M is typical of physiological levels found in circulation during youth and a concentration range from 1 × 10^−9^ to 1 × 10^−7^ M is frequently utilized under experimental conditions (44, 45). Furthermore, cultured human inflammatory cells, including U937 cells, have previously been utilized as *in vitro* models of estrogen-mediated aging (46, 47).

Nitric oxide production was measured in each treatment group (*n* = 12) by the Griess method (48) with the absorbance of a 1:1 ratio of sample and Griess reagent read using a Biotech Synergy HT plate reader at a wavelength of 540 nm. Concentrations were calculated against a standard curve of sodium nitrite standards (0, 1, 10, 25, 50, 75, and 100 µM).

The human iNOS sandwich-enzyme-linked immunosorbent assay (Elabscience, USA) was used to measure iNOS levels in each treatment group (*n* = 12) according to the manufacturer’s instructions and published work (49). Optical density readings were performed using a Biotech Synergy HT plate reader at a wavelength of 450 nm and concentrations were calculated against the standard curve generated within the assay.

### Statistical Analysis

Readings for NO and iNOS were measured in four replicate sample wells and average concentrations were determined from three independent experiments (*n* = 12 in total). Treatment groups were analyzed by ANOVA and *t*-tests (parametric data) or Mann–Whitney *U*-tests (non-parametric data) using SPSS (Version 22). A probability (*P*) value of *P* < 0.05 was considered statistically significant in all cases.

## Results

### Purification of CRP Isoforms

**Figure 2:**
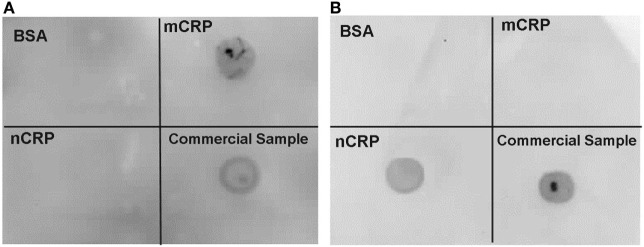
Immunoblot confirming purity of C-reactive protein (CRP) isoforms. Immunoblotting confirmed the presence of monomeric CRP (mCRP) alone **(A)** and native CRP (nCRP) alone **(B)** in purified samples. Both membranes included blots for a non-specific bovine serum albumin negative control protein, the purified mCRP sample, the purified nCRP, and the original commercial CRP sample. The mCRP and nCRP isoforms were detected with monoclonal anti-mCRP 8C10 [membrane **(A)**] and monoclonal anti-nCRP 1D6 [membrane **(B)**] respectively.

Immunoblotting confirmed the purity of CRP isoforms (Figure 2) used in the experiments. There was no detection of mCRP in the purified nCRP sample and no detection of nCRP in the purified mCRP sample. Purification yields for nCRP and mCRP were 4.5 and 2.3 mg/ml, respectively prior to normalization at 2 mg/ml.

Moreover, both the nCRP and mCRP samples at 2 mg/ml were confirmed effectively endotoxin-free (<20 ρg/ml), thus excluding LPS contamination in the experimental treatments. In essence, the deliberate pre-activation of some U937 cells with 0.5 mg/ml LPS exposed those cells to at least 25 million times more endotoxin than was present in normalized CRP samples, and at least 50 and 200 million times more endotoxin than was present in experimental concentrations of nCRP (<10 ρg/ml LPS) and mCRP (<2.5 ρg/ml LPS), respectively. Therefore, the negligible endotoxin levels in CRP treatments cannot account for observed experimental changes in NO.

### Flow Cytometry

**Figure 3:**
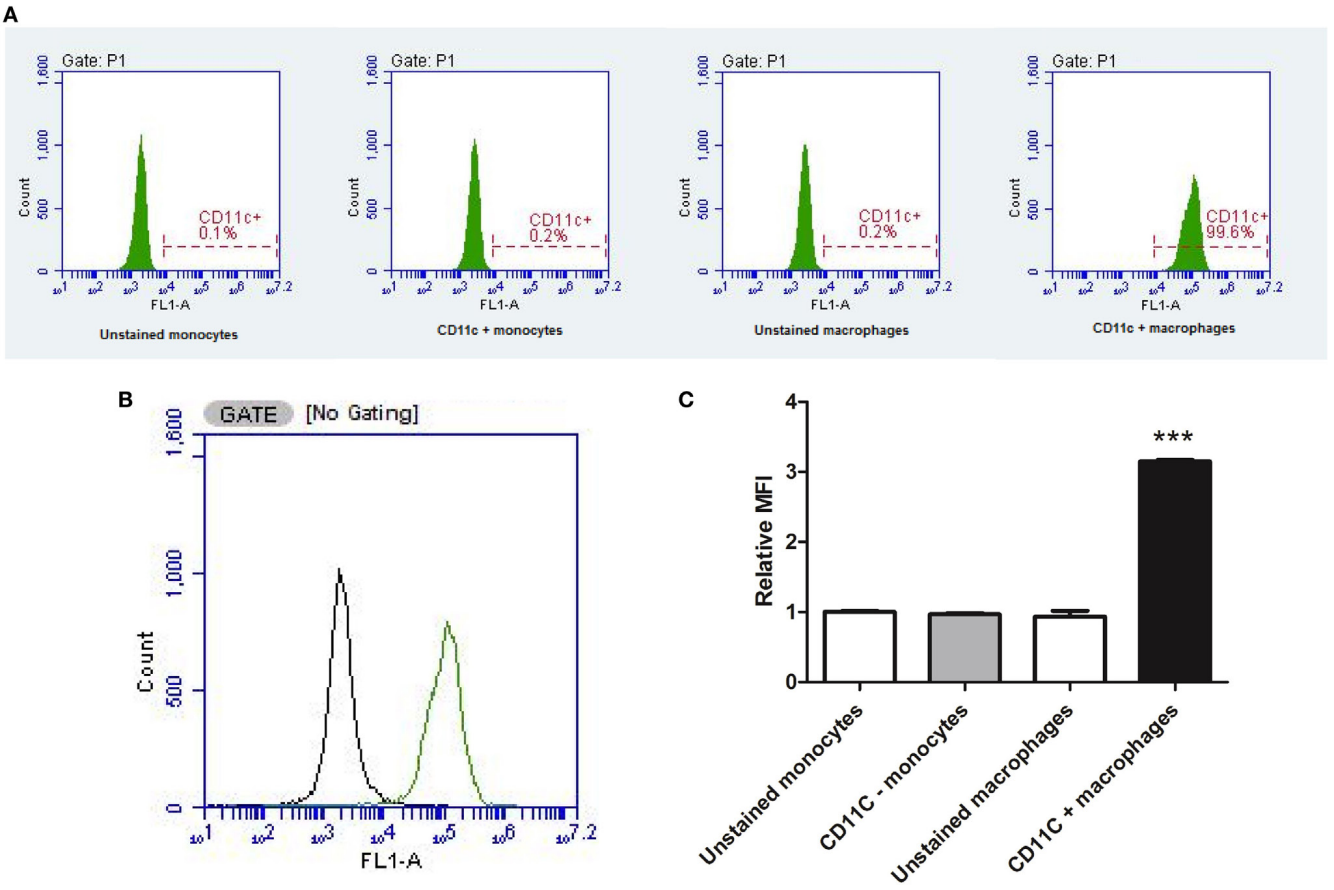
Flow cytometry confirming macrophage differentiation. The conversion of U937 monocytic cells into a distinct population of macrophage was confirmed *via* detection of the CD11c surface marker by flow cytometry (data represent an average of three independent experiments). Phorbol 12-myristate 13-acetate-differentiated cells almost exclusively expressed the CD11c macrophage marker, whereas monocytes predominantly lacked the CD11c surface marker **(A)**. Two distinct populations of cells were detected **(B)** with significantly higher median fluorescence intensity (MFI) in PMA-treated cells compared to undifferentiated U937 monocytes **(C)**. ***Indicates significant difference in MFI (*P* < 0.001; *n* = 3). Errors bars represent the SEM.

The conversion of U937 monocytic cells into a distinct population of macrophage was confirmed *via* detection of the CD11c surface marker by flow cytometry (Figure 3). PMA-differentiated cells almost exclusively (99.6% CD11c^+^) expressed the CD11c macrophage marker, whereas monocytes predominantly lacked the CD11c surface marker (0.2% CD11c^+^). The MFI was significantly (*P* < 0.001) higher in PMA-treated cells compared to undifferentiated U937 monocytes, confirming PMA transformed U937 monocytes into CD11c^+^ macrophages.

### The Effect of Exogenous CRP Isoforms on NO and iNOS Production

**Figure 4:**
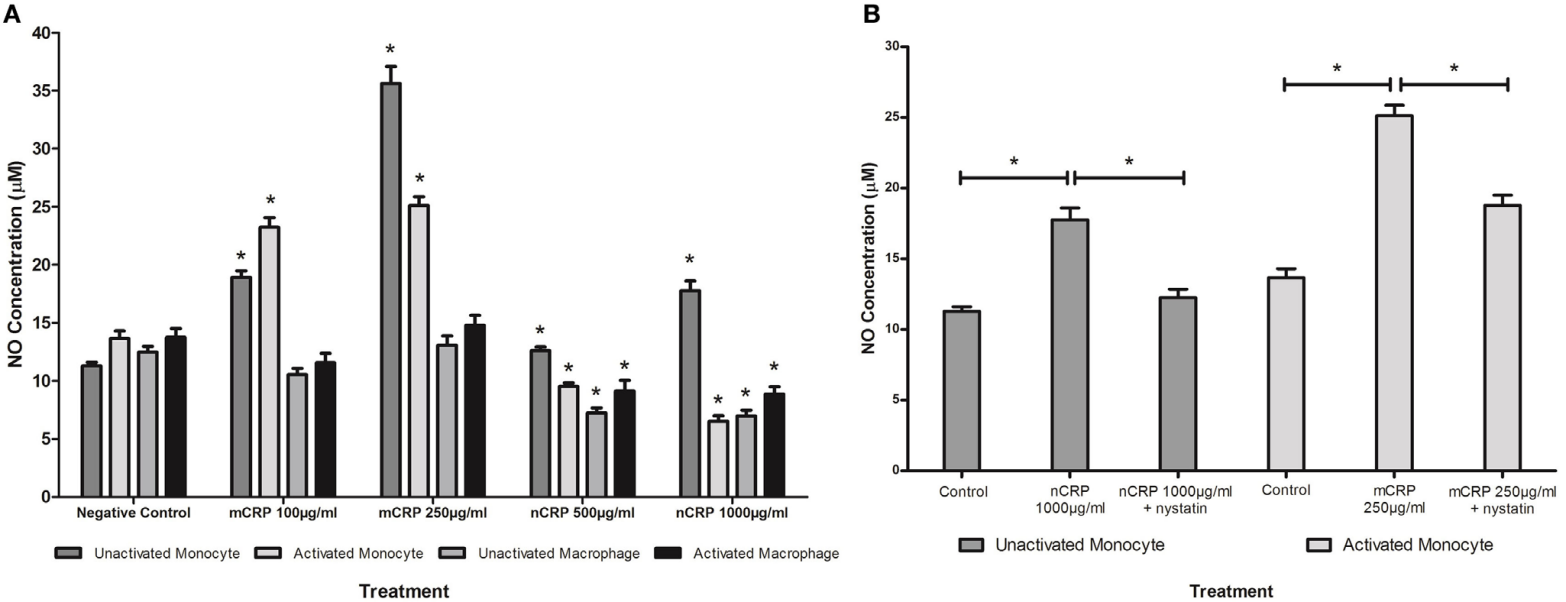
Effect of C-reactive protein (CRP) isoforms on nitric oxide (NO) production. NO production by several cell states (monocyte, lipopolysaccharide (LPS)-activated monocyte, macrophage, and LPS-activated macrophage) was differentially mediated by CRP isoforms in a concentration-dependent manner **(A)**. Nystatin significantly reversed CRP-mediated NO production in unactivated monocytes treated with native CRP (Ncrp) and activated monocytes treated with mCRP **(B)**. *Indicates significant differences (*P* < 0.05; *n* = 12) in NO production compared with corresponding negative controls **(A)** or CRP/nystatin treatments **(B)**. Errors bars represent the SEM.

**Figure 5:**
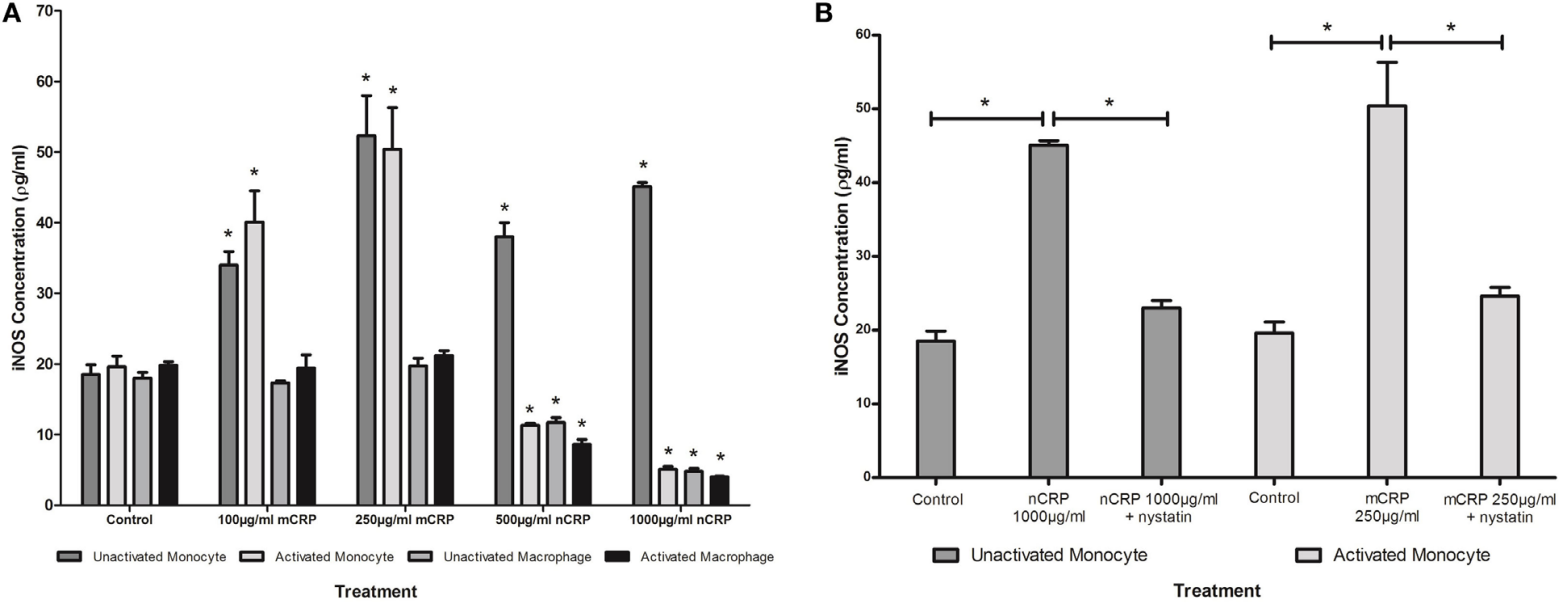
Effect of C-reactive protein (CRP) isoforms on inducible nitric oxide synthase (iNOS). Levels of iNOS production by several cell states (monocyte, lipopolysaccharide (LPS)-activated monocyte, macrophage, and LPS-activated macrophage) were differentially mediated by CRP isoforms in a concentration-dependent manner **(A)**. Nystatin significantly reversed CRP-mediated iNOS production in unactivated monocytes treated with nCRP and activated monocytes treated with mCRP **(B)**. *Indicates significant differences (*P* < 0.05; *n* = 12) in iNOS production compared with corresponding negative controls **(A)** or CRP/nystatin treatments **(B)**. Errors bars represent the SEM.

The NO values obtained in the study were in line with published findings utilizing unstimulated and LPS-activated U937 cells (50–52). Monocytic cells exhibited a substantial (more than 1.5-fold) concentration-dependent increase (*P* < 0.05) in NO and iNOS production when treated with mCRP (Figures 4A and 5A), regardless of their activation state. The mCRP isoform did not have any effect (*P* > 0.05) on NO or iNOS production by unactivated or LPS-activated macrophages, whereas nCRP significantly (*P* < 0.05) reduced NO and iNOS production by both unactivated and LPS-activated macrophages. However, the greatest reduction in NO (from 13.7 to 6.5 µM) and iNOS (from 19.6 to 5.1ρg/ml) was observed by LPS-activated monocytes following treatment with (1,000 µg/ml) nCRP. Interestingly, the nCRP isoform had the opposite effect in unactivated monocytes, significantly (*P* < 0.05) increasing NO and iNOS following treatment with nCRP, but more modestly than the mCRP isoform.

### The Effect of Nystatin on CRP-Induced NO and iNOS Production

Sample treatments that exhibited significant changes in NO production were repeated with the nystatin inhibitor to determine whether the CRP-mediated responses involved interaction with lipid rafts in the cellular membrane of monocytes/macrophages. Figures 4B and 5B show significant reversal of NO and iNOS production following inhibition with nystatin. Non-significant results (*P* > 0.05) were excluded from Figures 4B and 5B for clarity, including treatment with nystatin alone (NO = 12.3 ± 0.4 and 12.9 ± 0.4 μM; iNOS = 18.3 ± 1.5 and 20.0 ± 1.0 ρg/ml in unactivated and activated monocytes respectively).

When unactivated monocytes were treated with nCRP there was a significant increase (*P* < 0.05) in the production of NO and iNOS. When co-treated with nystatin there was a significant reversal in both NO (*P* = 0.001) and iNOS (*P* < 0.001) production by unactivated monocytes treated with 1,000 µg/ml nCRP.

Treatment of LPS-activated monocytes with mCRP induced a significant (*P* < 0.001) increase in NO and iNOS production. Again, nystatin significantly reversed the mCRP-induced NO (*P* < 0.05) and iNOS (*P* < 0.001) production by LPS-activated monocytes back to levels observed in the corresponding control.

### The Effect of Estrogen on CRP-Induced Nitric Oxide Production

**Figure 6:**
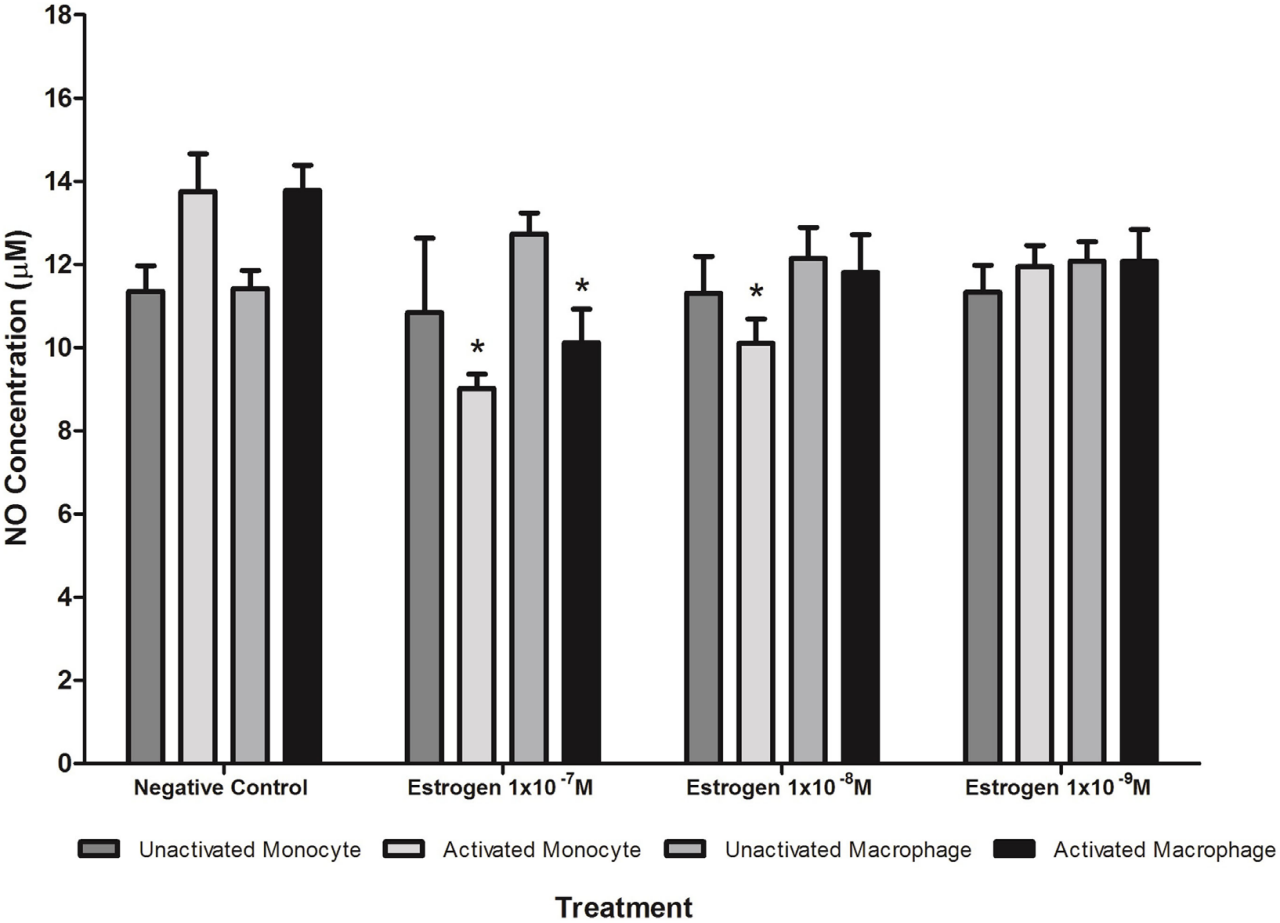
Effect of estrogen on nitric oxide (NO) production. Estrogen reduced NO production by lipopolysaccharide (LPS)-activated U937 monocytes and macrophages. * denotes a significant (*P* < 0.05; *n* = 12) difference in NO production followed by treatment with estrogen (1 × 10^−7^ 1 × 10^−8^, or 1 × 10^−9^ M) compared to the negative control. Errors bars represent the SEM.

**Figure 7:**
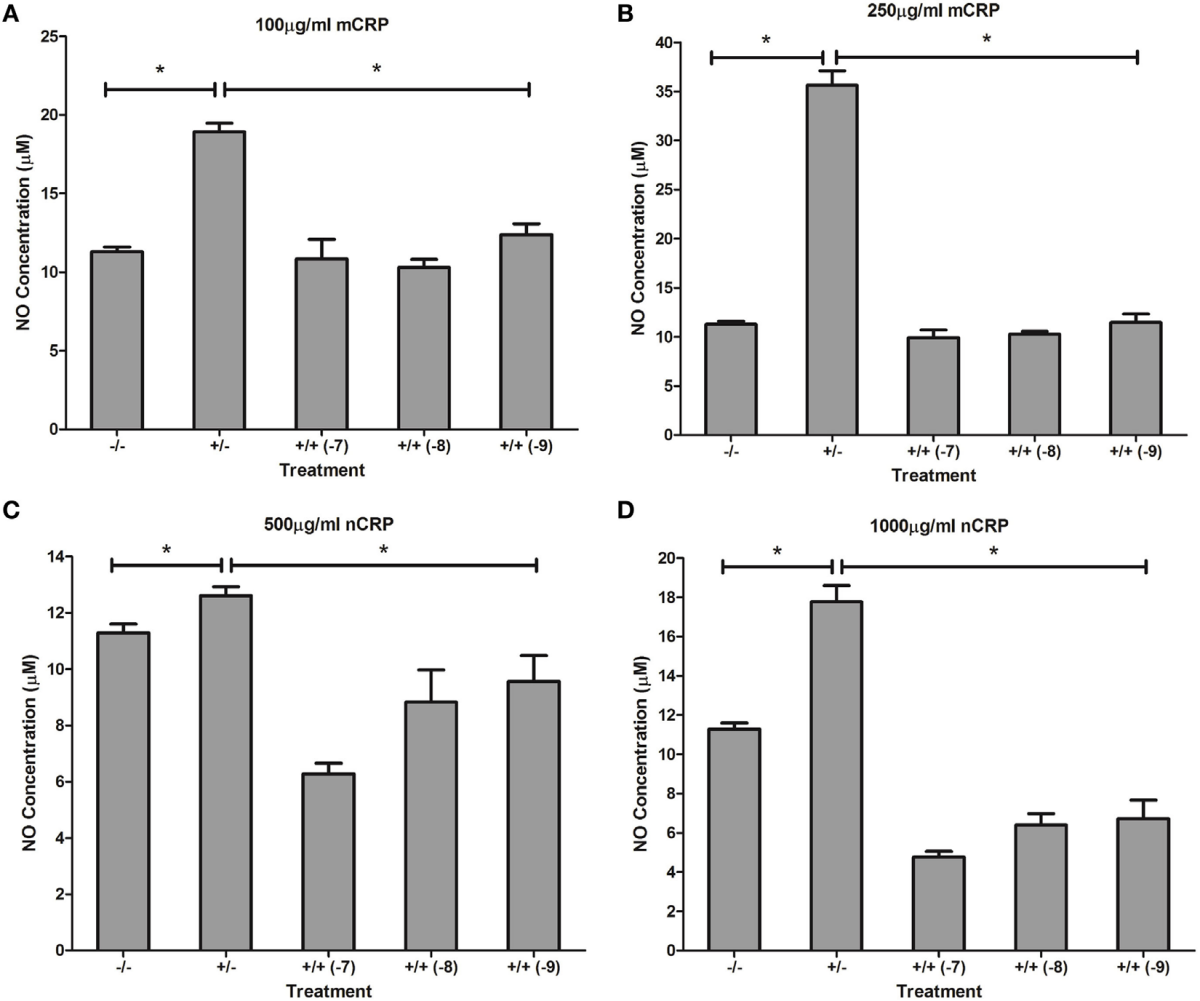
Effect of estrogen on C-reactive protein (CRP)-mediated nitric oxide (NO) production by unactivated U937 monocytes. Estrogen reversed CRP-induced NO production by unactivated U937 monocytes **(A–D)**. ^−/−^ represents the untreated negative control with no CRP or estrogen supplementation. ^+/−^ represents treatment with CRP alone. ^+/+^ represents treatment with both CRP and estrogen. The number in parentheses indicates the concentration (1 × 10^−7^, 1 × 10^−8^, or 1 × 10^−9^ M) of estrogen. *Denotes a significant (*P* < 0.05; *n* = 12) difference in NO production following treatment with CRP or co-supplementation with estrogen. Errors bars represent the SEM.

Treatment with estrogen controls in the absence of CRP isoforms (Figure 6) showed that physiological concentrations of estrogen reduced NO production by monocytes and macrophages exposed to endotoxin. Treatment of unactivated monocytes with either CRP isoform alone significantly (*P* < 0.001) increased NO production. However, all concentrations of estrogen co-supplementation significantly decreased (*P* < 0.01) CRP-induced NO production by unactivated monocytes (Figure 7) to levels similar or below those of the untreated control. Moreover, the reduction in NO by LPS-activated monocytes following treatment with estrogen alone (in the absence of CRP isoforms) was relatively modest compared to the estrogen-mediated reversal of NO production following co-incubation with mCRP.

**Figure 8:**
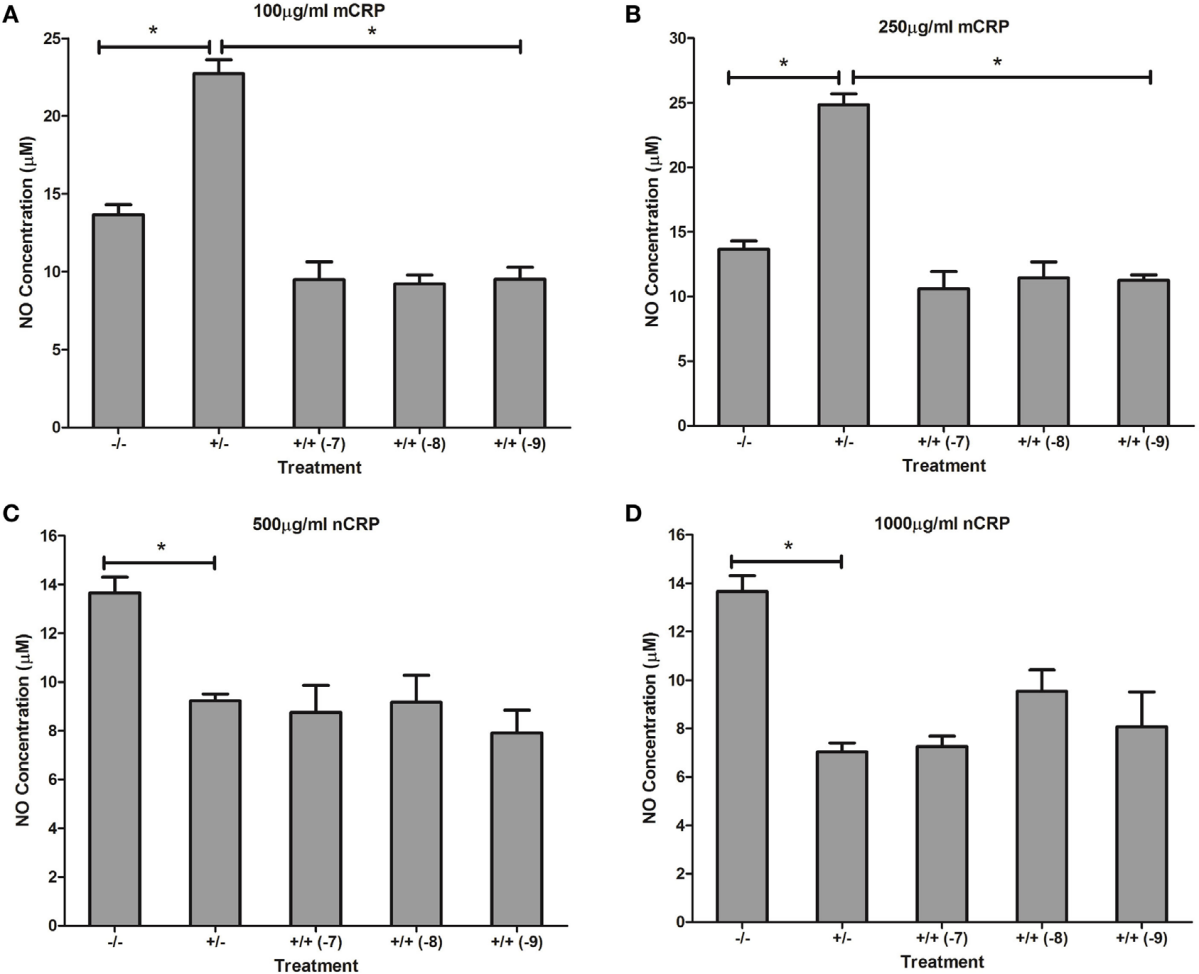
Effect of estrogen on C-reactive protein (CRP)-mediated nitric oxide (NO) production by lipopolysaccharide (LPS)-activated U937 monocytes. Estrogen reversed monomeric CRP (mCRP)-induced NO production **(A,B)** but had no effect on native CRP (Ncrp)-mediated inhibition of NO production **(C,D)** by activated U937 monocytes. ^−/−^ represents the untreated negative control with no CRP or estrogen supplementation. ^+/−^ represents treatment with CRP alone. ^+/+^ represents treatment with both CRP and estrogen. The number in parentheses indicates the concentration (1 × 10^−7^, 1 × 10^−8^, or 1 × 10^−9^M) of estrogen. *Denotes a significant (*P* < 0.05; *n* = 12) difference in NO production following treatment with CRP or co-supplementation with estrogen. Errors bars represent the SEM.

Similar to the response by unactivated monocytes, mCRP significantly (*P* < 0.001) increased NO production by LPS-activated monocytes. In direct contrast, there was a significant (*P* < 0.01) dose-dependent decrease in NO production by LPS-activated monocytes following treatment with nCRP. Estrogen significantly (*P* < 0.05) reversed mCRP-induced NO production to levels below that of the control while having no effect on the nCRP-mediated inhibition of NO production by LPS-activated monocytes (Figure 8).

**Figure 9:**
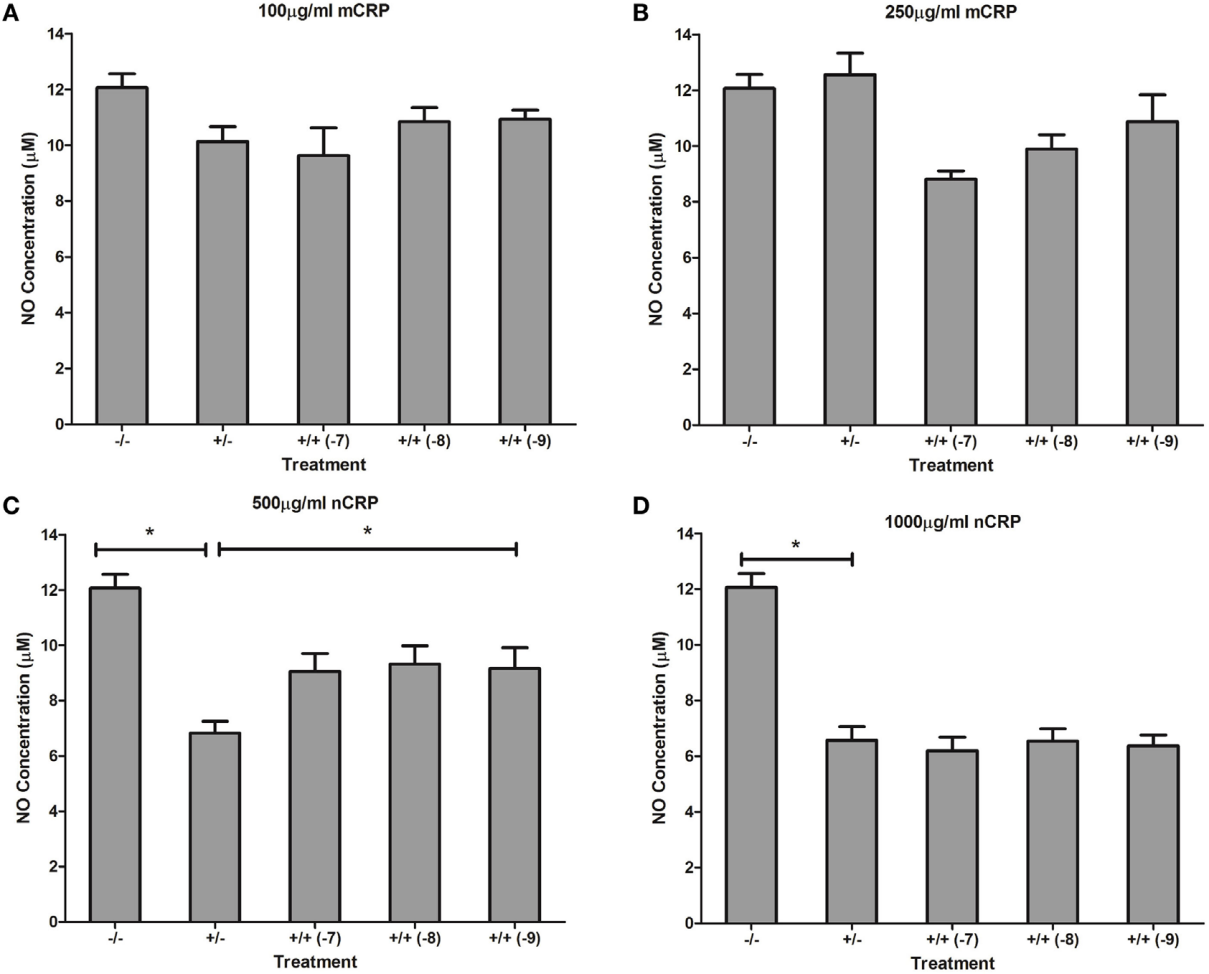
Effect of estrogen on CRP-mediated NO production by unactivated U937 macrophages. Estrogen had no effect on NO production when co-supplemented with mCRP in unactivated **(A,B)** macrophages. However, estrogen reversed the nCRP-mediated reduction in NO production by unactivated macrophages treated with low **(C)** but not high **(D)** levels of nCRP. ^−/−^ represents the untreated negative control with no CRP or estrogen supplementation. ^+/−^ represents treatment with CRP alone. ^+/+^ represents cells treatment with both CRP and estrogen. The number in parentheses indicates the concentration (1 x 10^−7^, 1 x 10^−8^, or 1 x 10^−9^M) of estrogen. * denotes a significant (*P* < 0.05; *n* = 12) difference in NO production following treatment with nCRP or co-supplementation with estrogen. Errors bars represent the SEM.

**Figure 10:**
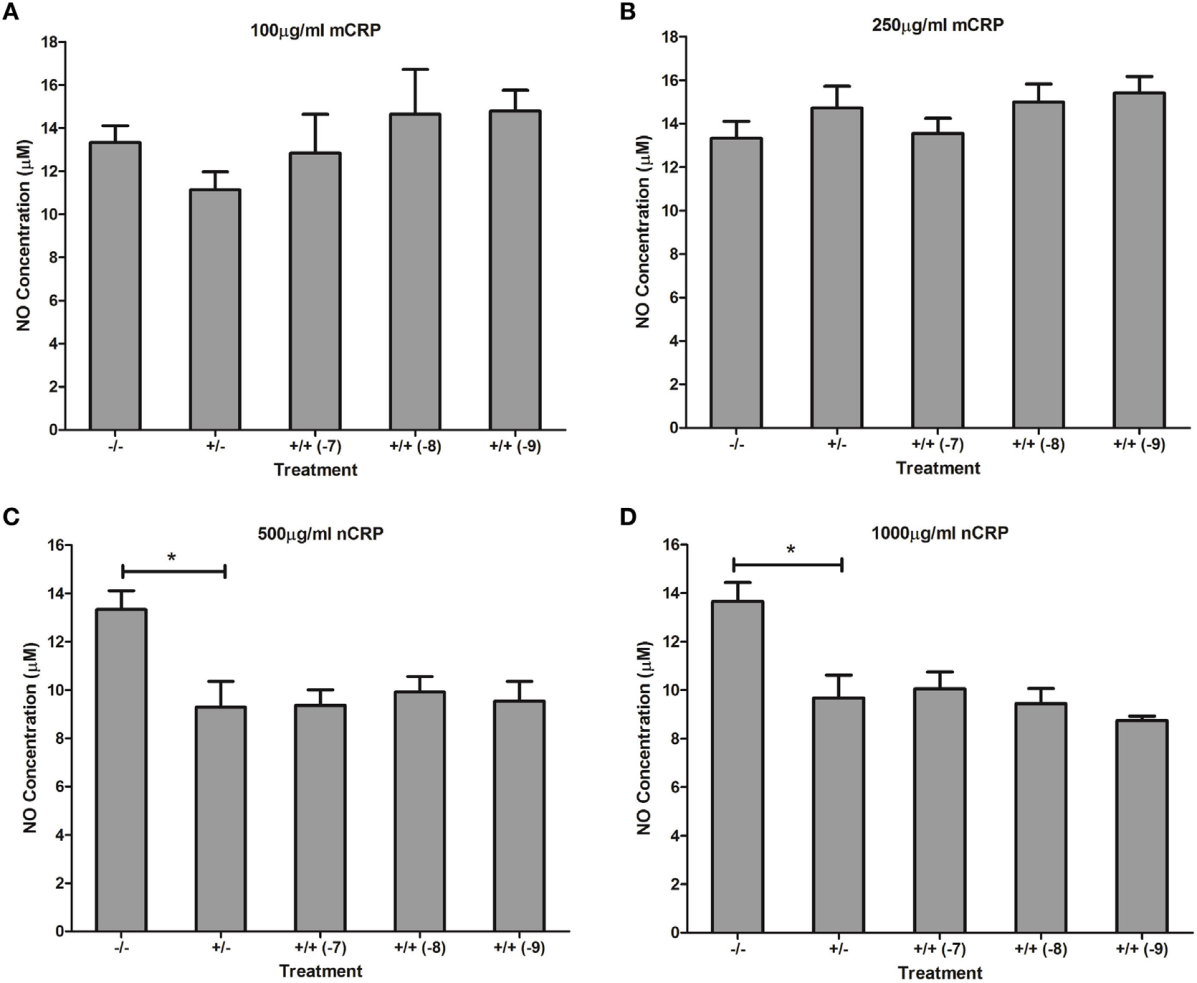
Effect of estrogen on CRP-mediated NO production by LPS-activated U937 macrophages. Estrogen had no effect on NO production when co-supplemented with mCRP **(A,B)** or nCRP **(C,D)** in activated U937 macrophages. ^−/−^ represents the negative control with no CRP or estrogen supplementation. ^+/−^ represents treatment with CRP alone. ^+/+^ represents treatment with both CRP and estrogen. The number in parentheses indicates the concentration (1 x 10^−7^, 1 x 10^−8^, or 1 x 10^−9^M) of estrogen. * denotes a significant (*P* < 0.05) reduction in NO production following treatment with nCRP compared to the control (*n* = 12). Errors bars represent the SEM.

Estrogen had no significant (*P* > 0.05) effect on NO production when co-supplemented with mCRP in unactivated (Figures 9A,B) and LPS-activated (Figures 10A,B) macrophages, mirroring the fact treatment with mCRP alone had no significant effect on NO production by macrophages.

The nCRP isoform significantly reduced NO production by unactivated and LPS-activated macrophages. Estrogen significantly reversed (*P* < 0.01) the nCRP-mediated reduction in NO production by unactivated macrophages treated with low (Figure 9C) but not high (Figure 9D) levels of nCRP. Co-incubation with estrogen maintained the nCRP-mediated reduction in NO production by LPS-activated macrophages (Figures 10C,D), in line with modest reductions in NO following treatment with estrogen alone (in the absence of CRP isoforms).

## Discussion

### The Effect of Exogenous CRP Isoforms on NO Production

The response to each CRP isoform appeared to be dependent on the differentiation and activation status of cells. The mCRP isoform stimulated NO and iNOS production by monocyte-like U937 cells, regardless of the activation state. This agrees with studies showing CRP can induce NO production in both the presence and absence of infection (28, 53). The mCRP isoform has previously been shown to stimulate NO production in neutrophils (34). In stark contrast, the nCRP isoform reduced NO and iNOS production by macrophage-like cells, both in the presence and absence of endotoxin. Furthermore, in LPS-activated monocytes, nCRP reduced NO and iNOS levels in a concentration-dependent manner. These findings support growing evidence that nCRP often has opposing actions to mCRP and dampens the overall inflammatory response relative to that induced by mCRP following tissue injury or infection (54, 55).

Collectively, these findings suggest that CRP isoforms mediate NO levels in U937 monocytes/macrophage *via* changes in the levels of iNOS. Previous work has also showed that CRP can mediate NO production in cardiac myocytes *via* iNOS (53) but responses to each specific CRP isoform were not determined. The data indicate that nCRP may have an inhibitory effect on NO and iNOS production by circulating monocytes following activation by bacterial endotoxin, and by tissue macrophages with or without prior LPS-activation. In direct contrast, the data suggest mCRP may have a stimulatory effect on NO and iNOS production by circulating monocytes with or without prior activation by bacterial endotoxin but this effect is lost once monocytes differentiate into tissue macrophages. In the absence of infection, the findings indicate both nCRP and mCRP increase NO and iNOS production by monocytes but the stimulation by nCRP is relatively modest compared to that produced by mCRP at CRP concentrations typical of an acute inflammatory response. The relative proportion of each CRP isoform *in vivo* could possibly influence the inflammatory response generated, with the formation of mCRP from the dissociation of nCRP promoting inflammation. Elevated levels of NO are known to induce the production of pro-inflammatory cytokines (56) including IL-6 and tumor necrosis factor alpha (TNF-α) by both monocytes/macrophages (57, 58) and neutrophils (59). The release of such cytokines can stimulate iNOS, leading to further NO production (60) and increased phagocytosis (61).

### Effect of Nystatin on CRP-Induced NO Production

Nystatin was the only inhibitor applied in this study. Several other inhibitors had been considered but could not be utilized, since they directly affect NO production (62–64). Nystatin is a relatively mild inhibitor that binds to cholesterol and disassembles lipid rafts in cellular membranes (65). The mCRP isoform is known to associate with lipid rafts in the cell membranes (13, 66).

In this study, nystatin reversed nCRP-induced NO and iNOS production by unactivated monocytes and mCRP-induced NO and iNOS production by LPS-activated monocytes. Thus, given nystatin disrupts lipid rafts and these findings suggest nCRP may interact with lipid rafts in monocytes in the absence of infection, whereas mCRP may associate with lipid rafts in monocytes following infection. Given the binding of mCRP to integrins αvβ3 and α4β1 influences its pro-inflammatory actions (67), antagonizing these integrins may help to elucidate mechanisms by which mCRP mediates NO production.

In both unactivated and activated macrophages, nystatin had no significant (*P* > 0.05) effect on the nCRP-mediated reduction in NO and iNOS. This suggests that once monocytes become mature macrophages, nCRP might no longer associate with lipid rafts. The mCRP isoform had no effect on NO or iNOS production by macrophages in the presence or absence of LPS, so nystatin inhibition was irrelevant.

Treatment of U937 monocytes and macrophages with nystatin alone had no significant effect on NO levels, suggesting this inhibitor did not directly disrupt NO pathways through its effect on cell membranes. Indeed, nystatin has been routinely utilized in various investigations of CRP activity and studies have confirmed it does not cause a functional disturbance of cellular cholesterol homeostasis (13, 68).

### Effect of Estrogen on CRP-Induced NO Production

Physiological concentrations of estrogen had distinct effects on CRP-mediated NO production, dependent on cell differentiation and activation state, agreeing with studies showing that estrogen can have both suppressive and stimulatory effects on iNOS and NO production (35). The decrease in CRP-induced NO levels by estrogen in unactivated monocytes treated with nCRP or mCRP supports evidence showing estrogen reduces the inflammatory response, including the production of NO/iNOS (37, 40). Estrogen reversed mCRP-induced NO production by LPS-activated monocytes, while the nCRP-mediated reduction in NO production was maintained by physiological concentrations of estrogen in LPS-activated monocytes. This supports previous work showing estrogen suppresses pro-inflammatory effects, including NO production (69, 70) and inflammatory cytokines such as IL-6 in activated monocytes (71). Indeed, treatment of LPS-activated monocytes with estrogen in the absence of CRP also reduced NO levels but the reduction was modest compared to the estrogen-mediated reversal of NO production following treatment with mCRP.

In both unactivated and LPS-activated macrophages, nCRP significantly decreased NO production, indicative of the relative anti-inflammatory properties of nCRP in tissues (11). Reversal of the nCRP-mediated reduction in NO production by estrogen in unactivated macrophages concurs with findings showing increased iNOS expression and NO production by peritoneal macrophages and splenocytes exposed to estrogen (35, 72). Furthermore, some studies have shown that estrogen can induce interferon-gamma (IFN-γ) secretion and subsequent NO production by macrophages (35, 73, 74). The nCRP-induced reduction in NO by activated macrophages was sustained by physiological concentrations of estrogen, concurring with the reduction in NO production following treatment of LPS-activated macrophages with estrogen in the absence of CRP isoforms. These findings agree with recent evidence showing estrogen reduces the inflammatory response, including NO production, in LPS-activated macrophages (75).

## Conclusion

In conclusion, the findings suggest that CRP isoforms are potential targets for mediating NO production by human monocytes and macrophages. Each CRP isoform induced a distinct profile of NO responses that was dependent on cell differentiation and activation status. In the presence or absence of endotoxin, nCRP had an inhibitory effect on NO/iNOS production in macrophages whereas mCRP had no effect. The mCRP had a stimulatory effect on NO/iNOS synthesis in monocytes, with or without LPS-activation. The nCRP isoform had opposing effects in monocytes, reducing NO/iNOS production following LPS-activation but stimulating NO by unactivated monocytes. These findings warrant further investigation to confirm the results in additional cell lines and *ex vivo* primary human leukocytes, together with the use of selective inhibitors to elucidate the pathways involved. Animal models of age-related impaired healing could assess the effect of CRP isoforms on *in vivo* NO production during aging. The predominant inhibition of NO/iNOS production by nCRP in all cell states except unactivated monocytes and the stimulation of NO/iNOS by mCRP in monocytes supports growing evidence that mCRP initiates a greater inflammatory response relative to nCRP. Furthermore, the findings highlight the importance of the age-related decline in hormones (notably estrogen) on CRP-mediated responses and have implications for therapeutic strategies aimed at the elderly. With the exception of NO stimulation by estrogen in unactivated macrophages treated with nCRP, the predominant reduction of CRP-induced NO levels by estrogen supports evidence that estrogen reduces the inflammatory response following injury and infection. Thus, therapeutic strategies that restore estrogen levels to those found in youth and promote the stability of nCRP and/or prevent the formation of mCRP from the dissociation of nCRP may reduce overall NO production in age-related inflammatory conditions.

## Author Contributions

NS and ME conducted the experimental work, analysed and interpreted the data, and prepared the paper. WG and MS contributed to the conception and design of the study and revising the paper. JA was involved with all aspects of the work including conception/design of the study, supervising experimental work, analysing and interpreting the data, and writing and revising the paper.

## Conflict of Interest Statement

The authors declare that the research was conducted in the absence of any commercial or financial relationships that could be construed as a potential conflict of interest.
