# Supplementary figures and images for: The Effect of C-Reactive Protein Isoforms on Nitric Oxide Production by U937 Monocytes/Macrophages
Source: Front Immunol. 2018 Jul 2;9:1500. doi: 10.3389/fimmu.2018.01500 (PMC6036124; doi:10.3389/fimmu.2018.01500)

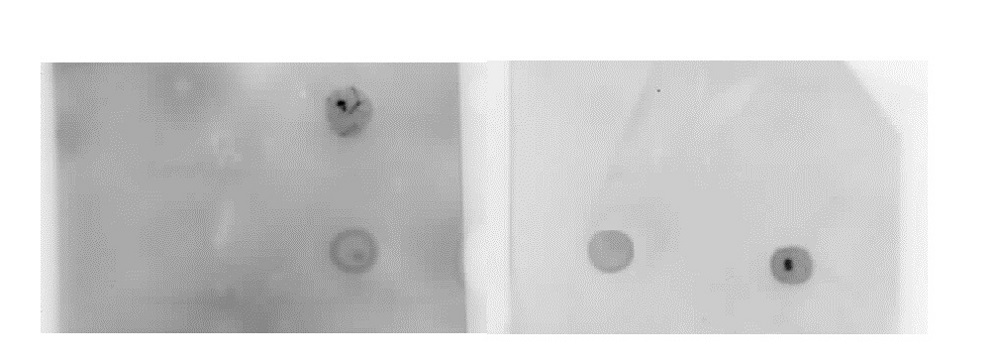

Supplement: Supplementary file 1 [file image_1.JPEG]
